# The longitudinal relationship between hearing loss and cognitive decline in tonal language-speaking older adults in China

**DOI:** 10.3389/fnagi.2023.1122607

**Published:** 2023-03-17

**Authors:** Xinxing Fu, Robert H. Eikelboom, Bo Liu, Shuo Wang, Dona M. P. Jayakody

**Affiliations:** ^1^Beijing Institute of Otolaryngology, Otolaryngology-Head and Neck Surgery, Beijing Tongren Hospital, Capital Medical University, Beijing, China; ^2^Centre for Ear Sciences, Medical School, The University of Western Australia, Crawley, WA, Australia; ^3^Ear Science Institute Australia, Subiaco, WA, Australia; ^4^Department of Speech-Language Pathology and Audiology, University of Pretoria, Pretoria, South Africa; ^5^Curtin Medical School, Curtin University, Bentley, WA, Australia; ^6^WA Centre for Health and Ageing, The University of Western Australia, Crawley, WA, Australia

**Keywords:** age-related hearing loss, cognitive impairment, tonal language, loneliness, mental health

## Abstract

**Introduction:**

Previous longitudinal studies indicate that hearing loss and cognitive impairment are associated in non-tonal language-speaking older adults. This study aimed to investigate whether there is a longitudinal association between hearing loss and cognitive decline in older adults who speak a tonal language.

**Methods:**

Chinese-speaking older adults aged 60 years and above were recruited for baseline and 12 month follow-up measurements. All participants completed a pure tone audiometric hearing test, Hearing Impaired-Montreal Cognitive Assessment Test (HI-MoCA), and a Computerized Neuropsychological Test Battery (CANTAB). The De Jong Gierveld Loneliness Scale was used to measure loneliness, and the 21-item Depression Anxiety Stress Scale (DASS-21) was used to measure aspects of mental health. Associations between baseline hearing loss and various cognitive, mental and psychosocial measures were evaluated using logistic regression.

**Results:**

A total of 71 (29.6%) of the participants had normal hearing, 70 (29.2%) had mild hearing loss, and 99 (41.2%) had moderate or severe hearing loss at baseline, based on mean hearing thresholds in the better ear. After adjusting for demographic and other factors, baseline moderate/severe audiometric hearing loss was associated with an increased risk of cognitive impairment at follow-up (OR: 2.20, 95% CI: 1.06, 4.50). When pure-tone average (PTA) was modeled continuously, an average difference of 0.24 in HI-MoCA scores for every 10 dB increase in BE4FA existed, and an average difference of 0.07 in the change of HI-MoCA scores in a 12 month period.

**Discussion:**

The results revealed a significant longitudinal relationship between age-related hearing loss and cognitive decline in this cohort of tonal language-speaking older adults. Steps should also be taken to incorporate hearing assessment and cognitive screening in clinical protocols for older adults 60 years and above in both hearing and memory clinics.

## Introduction

Hearing loss is considered the fourth greatest cause of disability in the world, according to the measured years lived with disability (YLDs) (Cunningham and Tucci, 2017). In China, about 50% of people in their seventh decade (60 to 69 years of age) and 80% of people aged 80 years or above experience hearing loss (Gong et al., 2018). Hearing loss affects effective communication, which can significantly impact daily life, causing feelings of loneliness, isolation, and frustration (Ciorba et al., 2012; Bennett et al., 2022; Jayakody et al., 2022). Increased risk of depression, anxiety, stress, and frailty are also associated with untreated hearing loss (Jayakody et al., 2018a; Lawrence et al., 2020; Tian et al., 2021). Longitudinal studies suggest a relationship between hearing loss and cognitive decline in non-tonal language speaking adults (Curhan et al., 2019). The PAQUID (Personnes Agées Quid) study, which included 3,670 community-dwelling persons aged 65 years and higher, found that self-reported hearing loss was independently related to accelerated cognitive decline over a 25 year period (Amieva et al., 2015).

The state of cognitive impairment covers a wide range of disorders, from subjective memory complaints and mild cognitive impairment to dementia (Caracciolo et al., 2014). Those with mild cognitive impairment are at a high risk of progressing to dementia (Gauthier et al., 2006). Given the fact that there are presently no disease-modifying treatments available for adults with dementia, a focus on risk factor reduction, particularly modifiable risk factors, is necessary (Livingston et al., 2017). Mid-life hearing loss is linked to an increased risk of dementia, contributing 8% of the potentially modifiable risk factors (Livingston et al., 2020).

Differences between the structure of tonal and non-tonal languages may result in different associations between hearing loss and cognitive decline. Changes in pitch (tone) at the monosyllabic level transmit the different lexical meanings of a word in tonal languages. Psychophysiological data suggests that a tonal language background is associated with enhanced overall cognitive ability (Bidelman et al., 2013). Furthermore, tonal languages have different speech spectrums than non-tonal languages (Chen et al., 2011). For example, the long-term average speech spectrum of Mandarin is clustered between 0.5 and 2 kHz, whereas in English the speech spectrum may reach a higher frequency range (Hu et al., 2019). Mandarin speakers may be less susceptible to high-frequency hearing loss than non-tonal language speakers as they age.

The association between untreated age-related hearing loss and cognitive impairment in Mandarin-speaking older adults has been supported by data from cross-sectional (Ren et al., 2019; Fu et al., 2021) and case-control studies (Hung et al., 2015). However, it is challenging to determine the direction of these associations from these investigations. The best method to investigate the relationship between hearing loss and cognitive impairment is by using prospective longitudinal studies in people without cognitive impairment, or knowing the level of cognitive impairment, at baseline (Ford et al., 2018).

Most of the cross-sectional and longitudinal cohort studies of cognition and hearing loss conducted with Chinese older adults have a few limitations regarding methods used to assess hearing and/or cognitive function. For example, self-reported hearing loss, not the audiometric measures of hearing, was utilized in most of the longitudinal studies to date (Chen and Lu, 2020; Gao et al., 2020). Evidence shows that older participants tend to underestimate their hearing impairment by self-report (Kamil et al., 2015), and therefore a standard audiometric test of hearing is a more accurate way to assess hearing sensitivity.

Furthermore, most of the cognitive assessments included verbally loaded scales or questionnaires that rely on the hearing ability of participants, e.g., the Mini-Mental State Examination (MMSE), and Montreal Cognitive Assessment (MoCA). As hearing loss can influence participants’ performance during cognitive assessments, it is essential to adopt non-verbal cognitive test materials when assessing the cognitive functions of elderly participants with hearing loss (Jayakody et al., 2018b). Furthermore, although both the MMSE and MoCA are useful to evaluate global cognitive functioning, they do not assess specific cognitive domains. A thorough assessment of more specific domains of cognitive function may supply more information on the mechanism between hearing loss and cognitive decline.

When compared to people with normal hearing, those with impaired communication abilities brought on by untreated hearing loss have a higher risk of experiencing negative mental and psychosocial effects. Hearing loss has been linked to depression (Lawrence et al., 2020), anxiety (Jayakody et al., 2018a), social isolation (Shukla et al., 2020) and emotional loneliness (Jayakody et al., 2022). Late-life depression and social isolation each contribute to 4% of the modifiable risk factors of dementia (Livingston et al., 2020). Hence it is reasonable to incorporate mental health as a covariate when analyzing the association between hearing loss and cognitive impairment.

This study explored the potential links between hearing loss and cognitive decline in a tonal language-speaking adult population based on data from a longitudinal study, utilizing both a non-verbal computer-based assessment battery for the different domains of cognitive functions (CANTAB) and a modified Montreal Cognitive Assessment for hearing impairment (HI-MoCA). We aimed to quantify the association between baseline hearing loss categories and cognitive impairment at follow-up, and also the association between audiometric hearing level and various domain-specific cognitive functions at follow-up.

## Materials and methods

This study’s protocol was previously published as part of a report of a cross-sectional study on hearing loss and cognitive performance in an adult Chinese population (Fu et al., 2021). These are summarized as follows.

### Participants

Participants were recruited through social media notices and community activities. Participants were native Mandarin speakers and at least 60 years of age when enrolled. Those who were not in general good health, unable to complete tasks necessary for the cognition evaluation tasks due to an underlying medical or mental illness, or who had previously worn or were using hearing aids or a hearing implant at the time of the study were excluded from the study.

The University of Western Australia-Human Research Ethics Committee (RA/4/20/5538) and Beijing Tongren Hospital, Capital Medical University (TRECKY2019-090) approved this study. Permission was also obtained from MoCA officials. All procedures were carried out in compliance with these approvals, and all participants signed informed consent forms.

### Hearing assessment

Hearing loss was assessed by pure-tone audiometry with an audiometer (Conera Audiometer, GN Otometrics Ltd, Denmark). For all participants, bilateral air-conduction thresholds were measured at 0.25, 0.5, 1, 2, 4, 6, and 8 kHz through a standard audiometric assessment conducted by a qualified audiologist in a soundproof booth at the audiology center of the Beijing Institute of Otolaryngology.

Two different methods were used to classify the hearing loss: the traditional average of the four mid-frequency hearing thresholds at 0.5, 1, 2, and 4 kHz in the better ear, and the average of the three high-frequency hearing thresholds at 4, 6, and 8 kHz in the better ear, noted respectively, as BE4FA and BE3HFA. Hearing thresholds at the higher frequencies were included because these frequencies contribute to the clarity of speech, whereas hearing in lower frequencies is necessary for speech comprehension. Furthermore, hearing loss in adults usually starts in the higher frequencies which may not be captured by summarizing the mid-frequencies. The hearing thresholds were also classified into a categorical variable, normal hearing (BE4FA ≤ 25 dB HL), mild hearing loss (26 to 40 dB HL), and moderate or above hearing loss (>40 dB HL) (Humes, 2019).

### Cognitive assessment

HI-MoCA was used to assess the individuals’ overall cognitive abilities. A Mandarin version of the MoCA (Version 7) was adapted, which was converted into a timed PowerPoint (Microsoft Corp., Redmond, WA, USA) presentation with all the verbal instructions replaced with visual instructions, here named HI-MoCA (Lin et al., 2017). To account for the learning effects, two alternative versions, v7.2 and v7.3, were adopted respectively, for baseline and follow-up assessments. All of the HI-MoCA tests were performed by an audiologist who received formal training from a senior neurology physician with extensive clinical experience and completed the MoCA certificated training course. A cutoff score of 26 was adopted to differentiate those with cognitive impairment from those who were cognitively normal (Nasreddine et al., 2005).

The evaluation of cognitive function for this study was focused on working and episode memory, processing speed, and special information processing. CANTAB is a neuropsychological test battery that was used to measure four non-verbal cognitive functions (Cambridge Cognition, Cambridge, UK): Motor Screening (MOT), delayed matching to sample (DMS), paired associates learning (PAL), and spatial working memory (SWM).

Motor screening screens visual, movement, and comprehension issues that could limit the data collection. The DMS test assesses both simultaneous visual matching ability and short-term visual recognition memory for non-verbalisable patterns (Owen et al., 1993). The percentage of correct patterns was the DMS outcome metric. The PAL test assesses episodic visuospatial memory, learning and association ability (Sahakian et al., 1988). PAL errors (total shapes) and PAL errors (six shapes) were analyzed in this study. The SWM test measures executive function and non-verbal visuospatial working memory (Summers and Saunders, 2012). Three main outcome measures were calculated in this study: SWM within errors, SWM between errors, and a SWM strategy score.

### Assessment of loneliness

The Mandarin version of the 6-item De Jong Gierveld Loneliness Scale (DJGLS) was used to measure loneliness, a validated tool to assess overall, emotional, and social loneliness (Leung et al., 2008; Yang et al., 2018; Fung et al., 2019). In this 6-item scale, three statements are made about emotional loneliness and three about social loneliness. The overall loneliness score ranges from 0 (no loneliness) to 6 (severe loneliness). Similarly, emotional and social loneliness are scored from 0 (no loneliness) to 3 (severe loneliness).

### Assessment of depression, anxiety, and stress

The validated Chinese version (Wang et al., 2016) of the 21-item Depression Anxiety Stress Scale (DASS-21) was used to assess the symptoms of depression, anxiety, and stress for the past 7 days (Lovibond and Lovibond, 1995). The seven elements of each scale are rated on a four-point scale. Depression, anxiety, and stress scores are calculated by adding the scores of each scale, and then doubled to determine the total score for each. Higher scores indicate higher levels of depression, anxiety or stress.

### Covariates

Demographic information, including age (years), sex, and education (primary school or below, middle school, high school, or postsecondary), was collected in 2019/2020 using a self-report questionnaire. Lifestyle characteristics, including longest occupation in life, residential arrangements (living with a spouse, children, or living alone), marital status (single, married, widowed, or divorced), and drinking and smoking habits (never, former, or current), were obtained.

Participants reported their history of diabetes mellitus and cardiovascular-related diseases (history of heart disease, high cholesterol, atherosclerosis, hypertension). These were chosen as covariates because they may increase the risk of hearing loss (Tan et al., 2018) and cognitive impairment (Saedi et al., 2016), thus potential confounders.

Leisure activities, which constitute a major portion of daily living and provide cognitive stimulation for older adults, were classified into recreational, intellectual, physical, and social categories (Leung et al., 2010). The participants were asked whether they engaged in any leisure activities using a leisure activity checklist. They were asked to specify the types of activities and the extent of engagement by reporting the weekly frequency and duration of their participation in each activity.

### Statistical analysis

Continuous variables at both baseline and follow-up were described as the mean ± standard deviation, and the categorical variables were described as numbers (percentage). A further series of comparisons of hearing thresholds, cognitive performance, loneliness, and mental health scores were analyzed among these two waves by chi-square test or Wilcoxon rank-sum test.

Associations between baseline hearing loss and outcome cognition impairment at follow-up were evaluated using the odds ratio (OR) and its corresponding 95% confidence intervals (95% CI) by multiple logistic regression, adjusting for confounding variables, including age, sex, education level, smoking, drinking, marital status, chronic disease, and leisure activities. Odds ratios (OR) > 1.00 indicated increased odds of cognitive impairment with every unit increase in HL across participants and other variables, whereas OR < 1.00 indicated lower odds of those variables.

As a longitudinal study, the linear mixed-effect model (LME) was used, including the covariates listed above, to study the time to incident cognitive decline. The LME model is designed to analyze longitudinal data. This model offers the advantage of considering multiple observations within a subject and intrasubject correlation. It also allows for adjusting numerous potential confounding variables (Amieva et al., 2015). An interaction term between hearing loss and time was included in this model, to test the effect of baseline hearing loss on cognitive decline (the change of the cognitive scores during the 12 month’s follow-up).

Multivariate linear regression (forward stepwise) analyses were used to assess the associations between cognition and hearing loss, loneliness and DASS-21 (continuous variables). A regression coefficient and its 95% CI were calculated. The different CANTAB modules (DMS, PAL, and SWM scores) and HI-MoCA scores were entered as dependent variables, respectively. The collinearity tests were examined during each stepwise regression analysis. All statistical analyses were performed using Stata version 17 (StataCorp, College Station, TX). The significance level was *p* ≤ 0.05 based on two-sided tests.

## Results

Of the 293 patients recruited in 2018/2019 at baseline, 39 did not attend the 12 month follow-up and 14 had incomplete covariate information. These 53 were excluded, leaving 240 participants who participated in both baseline and follow-up waves.

**FIGURE 1:**
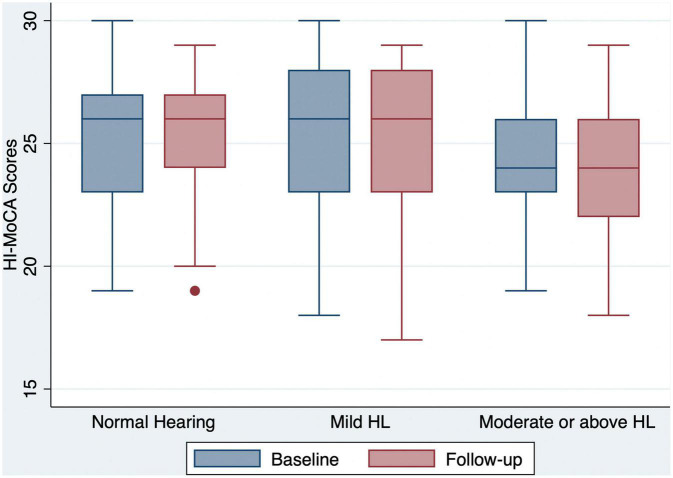
Box and whisker plot of HI-MoCA scores and quartiles at baseline and follow-up, according to the HL categories. One outlier for a participant with normal hearing at follow-up is shown by a dot. HI-MoCA, hearing impaired-Montreal cognitive assessment; HL, hearing loss.

**FIGURE 2:**
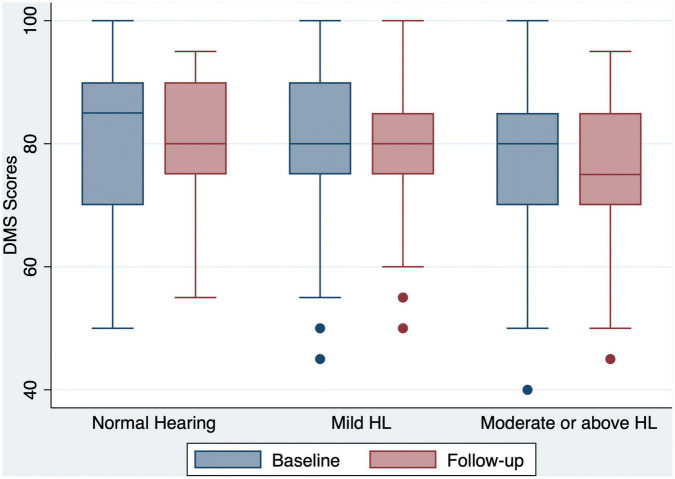
Box and whisker plot of DMS correct percent and quartiles at baseline and follow-up, according to the HL categories. Any outlier at baseline/follow-up is shown by a dot. DMS, delayed matching to sample; HL, hearing loss.

**FIGURE 3:**
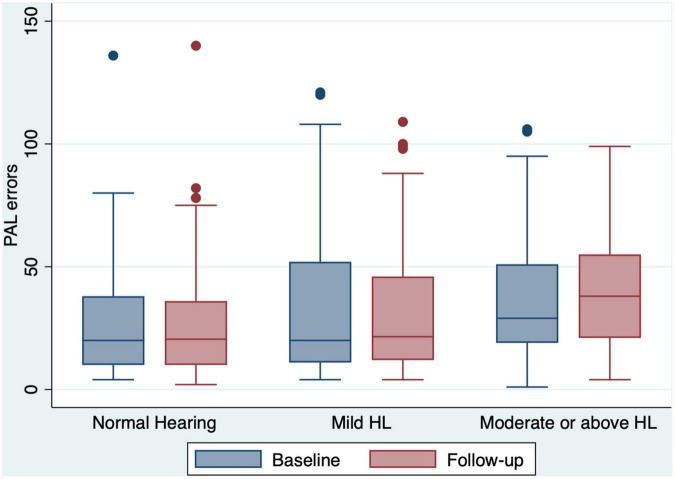
Box and whisker plot of PAL errors and quartiles at baseline and follow-up, according to the HL categories. Any outlier at baseline/follow-up is shown by a dot. PAL, paired associates learning; HL, hearing loss.

**FIGURE 4:**
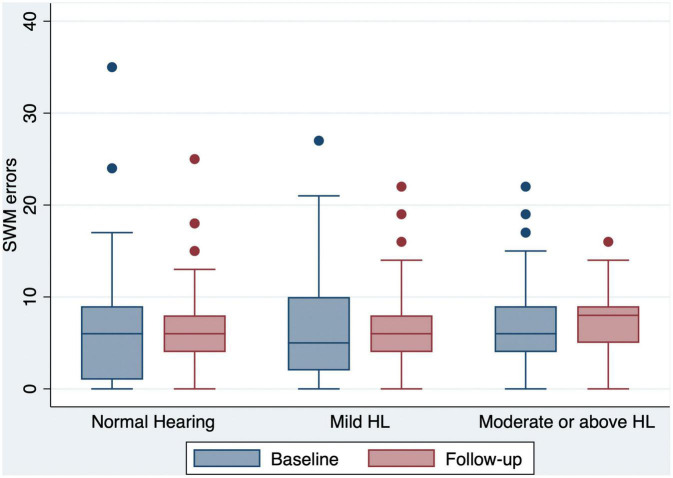
Box and whisker plot of SWM errors and quartiles at baseline and follow-up, according to the HL categories. Any outlier at baseline/follow-up is shown by a dot. SWM, spatial working memory; HL, hearing loss.

Of the 240 participants, 71 (29.6%) had normal hearing, 70 (29.2%) had mild hearing loss, and 99 (41.2%) had moderate or greater hearing loss at baseline (Table 1). Compared to the baseline scores, both the global and domain-specific cognitive scores, loneliness scales and mental health scores showed a significant decrease at the 12 month follow-up (Figures 1-4). Based on the Wilcoxon rank-sum test, a significant decline in HI-MoCA scores was seen (mean change = −0.30, *Z* = 6.713, *p* < 0.001). The mean progression of hearing loss was 0.68 and 1.58 dB/year for BE4FA and BE3HFA, respectively. Regarding the mental health and loneliness scores, we also observed significant changes over the follow-up period. The average change of depression, anxiety and stress scores were 0.83, 1.19, and 0.9, respectively (Supplementary Figure 1). Mean changes in loneliness scores were 0.13 and 0.20 points for emotional and social loneliness respectively, over 12 months (Supplementary Figure 2).

**TABLE 1:** Characteristics of the participants (*n* = 240) at baseline and 12 month follow-up.

Variables	Baseline	Follow up	Statistics values	Sig.
**Sex**				
Females *N, %*	145 (60.4)	n.a.	−	−
Males *N, %*	95 (39.6)	n.a.	−	−
Age (years) *Mean, SD*	69.7 (4.6)	70.7 (4.6)	−	−
60−64 years *N, %*	22 (9.2)	14 (5.8)	−	−
65−69 years *N, %*	126 (52.5)	102 (42.5)		
70−74 years *N, %*	66 (27.5)	92 (38.3)		
75 + years *N, %*	26 (10.8)	32 (13.3)		
**Education**				
Primary School *N, %*	14 (5.8)	n.a.	−	−
Middle School *N, %*	110 (45.8)	n.a.	−	−
College or above *N, %*	116 (48.3)	n.a.	−	−
**Hearing**				
BE4FA *Mean, SD*	39.6 (19.3)	40.3 (20.1)	*Z* = −6.415	<0.001
BE3HFA *Mean, SD*	56.1 (21.9)	57.6 (21.9)	*Z* = −10.670	<0.001
**HL Classification**				
Normal *N, %*	71 (29.6)	71 (29.6)	*x*^2^ = 436.512	<0.001
Mild *N, %*	70 (29.2)	67 (27.9)		
Moderate and above *N, %*	99 (41.2)	102 (42.5)		
**Cognitive scores**				
HI-MoCA *Mean, SD*	25.0 (2.6)	24.7 (2.7)	*Z* = 6.713	<0.001
Cognitive impairment *N, %*	99 (42.4)	105 (43.8)	*x*^2^ = 240.000	<0.001
SWM errors *Mean, SD*	6.7 (5.6)	7.0 (3.8)	*Z* = −2.926	0.003
PAL errors *Mean, SD*	34.1 (27.3)	35.2 (31.9)	*Z* = −4.107	<0.001
DMS correct percent *Mean, SD*	78.9 (11.8)	77.7 (11.2)	*Z* = 4.143	<0.001
**Medical history**				
Diabetes *N, %*	54 (22.6)	n.a.	−	−
Vascular disease *N, %*	189 (78.8)	n.a.	−	−
**Other**				
Smoking *N, %*	93 (38.8)	n.a.	−	−
Alcohol use *N, %*	78 (32.3)	n.a.	−	−
Divorced or singled *N, %*	26 (10.1)	n.a.	−	−
Living alone *N, %*	13 (5.4)	n.a.	−	−
Recreation activities *Mean, SD*	14.3 (10.0)	n.a.	−	−
Cognition activities *Mean, SD*	20.3 (12.5)	n.a.	−	−
Physical exercise *Mean, SD*	20.6 (11.1)	n.a.	−	−
Social activities *Mean, SD*	2.0 (2.6)	n.a.	−	−
**Loneliness**				
Emotional loneliness *Mean, SD*	0.9 (0.8)	1.1 (0.8)	*Z* = −5.191	<0.001
Social loneliness *Mean, SD*	1.1 (1.1)	1.3 (1.0)	*Z* = −4.976	<0.001
Overall loneliness *Mean, SD*	2.1 (1.6)	2.3 (1.5)	*Z* = −5.263	<0.001
**Mental health**				
Depression *Mean, SD*	3.7 (5.4)	4.6 (5.4)	*Z* = −8.828	<0.001
Anxiety *Mean, SD*	6.1 (5.5)	7.3 (4.6)	*Z* = −10.987	<0.001
Stress *Mean, SD*	6.6 (6.8)	7.6 (6.1)	*Z* = −10.373	<0.001

BE4FA, four frequencies (0.5, 1, 2, and 4 kHz) average of pure-tone hearing thresholds of the better ear; BE3HFA, three high-frequency (4, 6, and 8 kHz) average of pure-tone hearing thresholds of the better ear. HL, hearing loss; HI-MoCA, hearing impaired-Montreal cognitive assessment; Cognitive impairment, HI-MoCA < 26; PAL, paired associates learning; DMS, delayed matching to sample; SWM, spatial working memory.

Participants with hearing impairment at baseline had a higher incidence of developing mild cognitive impairment based on HI-MoCA cutoff scores, than those with normal hearing. The 12 month unadjusted OR of developing mild cognitive impairment was 3.23 (95% CI = 1.69−6.17) in subjects with moderate or above hearing loss (Table 2), but for participants with mild hearing loss, there was no significant risk of developing mild cognitive impairment. After adjusting for age, sex, and education, the risk of mild cognitive impairment was significantly higher in participants with baseline moderate or above hearing loss (OR = 2.2, 95% CI = 1.13−4.60) than in those with normal hearing. With further adjustment for additional covariates, including behavioral factors and health conditions such as smoking, alcohol, diabetes, and vascular disease, the association between moderate or above hearing loss and 12 month risk of mild cognitive impairment continued to be statistically significant (OR = 2.20, 95% CI = 1.06−4.50).

**TABLE 2:** Multivariable adjusted ORs and 95% CIs of the association between baseline hearing loss and 12 month cognitive impairment.

Hearing loss categories (Baseline)	Model 1 unadjusted	Model 2 age, sex, education adjusted	Model 3 multivariable adjusted
	**Odds ratio (95% confidence interval)**		
Normal hearing	Reference	Reference	Reference
Mild HL	1.50 (0.74, 3.01)	1.27 (0.60, 2.70)	1.23 (0.56, 2.70)
Moderate or above HL	3.23 (1.69, 6.17)	2.28 (1.13, 4.60)	2.20 (1.06, 4.50)

HL, hearing loss.

These results were confirmed when HI-MoCA scores were analyzed as a continuous variable. Table 3 shows the results of the linear mixed-effect model (Model 1) assessing the association between HI-MoCA scores and hearing loss and time. A lower HI-MoCA score was significantly associated with BE4FA (*B* = −0.04, *P* < 0.001) and 12 month follow-up period (*B* = −0.25, *p* < 0.001). Model 2 examined the association between hearing loss and cognitive decline, adjusted for age, sex, and educational level. The strength of the association between HI-MoCA scores and BE4FA and time decreased but was still significant (*B* = −0.03, and −0.23, *p* < 0.001, respectively). Finally, Model 3 assessed the same relationship as Model 2, with additional adjustments for several factors (mental health symptomatology, loneliness variables, leisure activities, and comorbidities). After controlling for these factors, the coefficients of cognitive decline with hearing loss and the follow-up period were still significant (*B* = −0.03, *p* = 0.002, and *B* = −0.16, *p* = 0.007, respectively). When BE3HFA was used as the independent variable, it was also significantly associated with the coefficients of cognitive decline in the three models.

**TABLE 3:** Relationship between the changes of averaged hearing thresholds and HI-MoCA scores over 12 months.

Variables	Model 1 unadjusted			Model 2 age, sex, education adjusted			Model 3 multivariable adjusted		
	**Beta**	**Sig.**	**R^2^**	**Beta**	***p***	**R^2^**	**Beta**	**Sig.**	**R^2^**
Time	−0.25	<0.001	0.20	−0.23	<0.001	0.19	−0.16	0.007	0.29
BE4FA	−0.04	<0.001		−0.03	<0.001		−0.03	0.002	
Time	−0.27	<0.001	0.18	−0.24	<0.001	0.18	−0.16	0.007	0.29
BE3HFA	−0.03	<0.001		−0.02	0.015		−0.02	0.024	

BE4FA, four frequencies (0.5, 1, 2, and 4 kHz) average of pure-tone hearing thresholds of the better ear; BE3HFA, three high-frequency (4, 6, and 8 kHz) average of pure-tone hearing thresholds of the better ear.

We conducted additional analyses to investigate the association between hearing loss and specific cognitive domains (short-term visual memory, learning ability, and executive function). When pure-tone average (PTA) was modeled continuously, we estimated an average change of 0.24 in HI-MoCA scores for every 10 dB increase in BE4FA and an average change of 0.07 in the change of HI-MoCA scores in a 12 month period for every 10 dB increase in BE4FA. As shown in Table 4, significant coefficients in the baseline scores of DMS and PAL with every 10 dB change were observed (*p* = 0.041 and 0.037), but not for SWM scores (*p* = 0.082). However, no significant changes in any of the specific CANTAB cognitive domain scores during the 12 month period were observed to be associated with hearing loss (continuous or categorical).

**TABLE 4:** Multivariable adjusted regression change over 12 months of cognitive performance by baseline hearing loss.

Variables	Baseline performance		Change over 12 months	
	**B (95% CI)**	**Sig.**	**B (95% CI)**	**Sig.**
**HI-MoCA**				
Mild HL	−0.10 (−0.81, 1.00)	0.833	−0.24 (−0.43, −0.04)	0.016
Moderate or above HL	−0.63 (−1.49, −0.23)	0.148	−0.52 (−0.72, −0.34)	<0.001
Every 10 dB	−0.24 (−0.42, −0.06)	<0.001	−0.07 (−0.10, −0.03)	<0.001
**DMS**				
Mild HL	−0.21 (−3.67, 3.24)	0.903	−0.98 (−2.45, 0.71)	0.183
Moderate or above HL	−5.81 (−10.0, −1.55)	0.008	−1.32 (−2.71, −0.06)	0.061
Every 10 dB	−0.88 (−1.72, −0.04)	0.041	−0.29 (−0.59, 0.01)	0.052
**PAL**				
Mild HL	0.12 (−9.27, 9.52)	0.980	0.16 (−0.90, 1.22)	0.768
Moderate or above HL	0.75 (−1.91, 3.43)	0.578	0.33 (0.67, 1.35)	0.509
Every 10 dB	0.60 (0.03, 1.17)	0.037	0.03 (−0.18, 0.25)	0.758
**SWM**				
Mild HL	0.25 (−1.79, 2.29)	0.808	−0.55 (−1.90, 0.79)	0.416
Moderate or above HL	0.38 (−1.55, 2.32)	0.698	0.02 (−1.26, 1.29)	0.979
Every 10 dB	0.36 (−0.04, 0.78)	0.082	0.03 (−0.30, 0.24)	0.818

HI-MoCA, hearing impaired-Montreal cognitive assessment; PAL, paired associates learning; DMS, delayed matching to sample; SWM, spatial working memory; HL, hearing loss.

## Discussion

Our results demonstrate that baseline hearing loss is associated with an increased risk of cognitive impairment at 12 months, as assessed by specific and general cognitive tests. However, the findings from this tonal language-speaking cohort do not appear to be significantly different from similar studies with non-tonal language speakers. Baseline moderate or severe hearing loss is associated with a greater risk of cognitive impairment after a 12 month follow-up period (OR: 2.20, 95% CI: 1.06, 4.50), compared with participants with normal hearing, after adjusting demographic and other factors, including mental health, loneliness status, behavior factors, and health conditions. Besides the general cognitive performance, an association was also observed between hearing loss and some specific baseline cognitive functions, including simultaneous visual matching ability and short-term visual recognition memory (DMS module in CANTAB) and episodic visuospatial memory (PAL module in CANTAB). This finding is consistent with previous longitudinal studies on the relationship between hearing loss measured by pure tone audiometry and cognitive impairment (Fischer et al., 2016), and cognitive decline (Deal et al., 2017) over time, in non-tonal languages speaking populations. In one of these previous studies, hearing impairment was independently associated with cognitive impairment over 10 years (hazard ratio: 1.90, 95% CI: 1.11, 3.26) in 1,884 subjects with a mean age of 66.7 years (Fischer et al., 2016). In the other study with 3,075 community-dwelling older adults over 9 years, audiometric hearing loss was related to lower baseline cognitive performance in memory, especially in participants with moderate or severe hearing loss (hazard ratio: 1.55, 95% CI: 1.10, 2.19) (Deal et al., 2017).

Baseline hearing loss was demonstrated to be a predictor of the change in overall cognitive performance (HI-MoCA) over 12 months, regardless of whether the continuous or categorical values were used. Furthermore, an association in the baseline scores of DMS and PAL with every 10 dB change of hearing loss was observed. These findings accord with those of previous studies, suggesting that these participants are at risk of developing dementia (Juncos-Rabadán et al., 2014; Campos-Magdaleno et al., 2021). However, no associations were observed between the rate of changes in any specific domains of cognitive functions over the 12 month follow-up period. This result is contrary to previous studies that demonstrated a faster cognitive decline in participants with hearing loss. For example, results from a 6 year follow-up study showed that a change in audiometric hearing acuity predicted changes in memory performance (Valentijn et al., 2005). Another study showed that individuals with baseline hearing loss had significantly faster annual rates of decline in the executive function (Lin et al., 2013). One possible explanation is that this study had a short follow-up period, only 12 months. It may take a longer time for hearing loss to show effects in specific cognitive domains (as examined in this study using the CANTAB modules). According to the sensory-deprivation mechanisms underlying the link between age-related hearing loss and cognitive decline, hearing and speech perception declines will cause cognitive declines due to the chronic reallocation of “high-level” cognitive resources, which may result in long-term changes in cognitive function (Wayne and Johnsrude, 2015).

Our findings are in general agreement with the findings in studies with non-tonal language speakers. Therefore, we provide no evidence to support the notion that speaking a tonal language provides an advantage over non-tonal language speakers over a 12 month period.

There is only a limited number of longitudinal studies in this field in tonal language speaking populations (Su et al., 2017). Most of the studies have been based on the analysis of data from the CLHLS (Chinese Longitudinal Healthy Longevity Survey), one of the largest cohort studies in China. However, it was not specially designed for the research on hearing or cognitive impairment, and only a self-reported hearing loss and a cognitive impairment screening tool (mini-mental state examination, MMSE) were utilized. This poses a potentially imprecise measurement of hearing and cognitive impairment. Su et al. reported that older adults with hearing loss were at a higher risk of subsequent dementia, HR 1.30 (95% CI: 1.14, 1.49), based on a cohort with 8,121 participants over 10 years of follow-up in Taiwan (Su et al., 2017). In another study conducted in Hong Kong, poor hearing at baseline was significantly associated with an increased risk of subjective memory complaints 12 months later, with an OR of 2.2 (95% CI: 1.8, 2.8) (Yu and Woo, 2019). To our knowledge, this current study is the first longitudinal study in mainland China utilizing a behavioral pure tone audiometry and non-verbal cognitive assessment tool.

In this study, we also observed a significant decrease in HI-MoCA scores over 12 months, with a mean change of 0.35 points in the cognitive impairment group and 0.21 points in the cognitively intact group. A previous study on the changes in MoCA scores over time, reported that a group with mild cognitive impairment at baseline showed a decline of 0.47 points/year over 3.5 years, while the cognitively intact group showed a decline of 0.18 points/year, but there was no significant difference between the two groups (Krishnan et al., 2017). We also revealed a decline rate of hearing level per year, 0.68 and 1.58 dB for BE4FA and BE3HFA, which is higher than previous findings on the progression of hearing loss in an ageing population, where the mean progression of hearing loss was 0.29 and 1.35 dB/year, respectively for low and middle frequencies (0.5, 1, 2, and 4 kHz), and high frequencies (2, 4, and 8 kHz) (Rigters et al., 2018).

Significant changes were observed in the mental health and loneliness scores over the follow-up period. One potential explanation for the significant changes in DASS-21 and loneliness scales, could be related to the impact of COVID-19. China was the first country that identified the coronavirus disease in the world at the end of 2019, and Beijing reported the first cases in February 2020 (Tian et al., 2020), just after the baseline data collection was collected. The follow-up data collection finished around the end of 2020 and the beginning of 2021, and several community-scale lockdowns were imposed by the local government between the baseline and follow-up sessions (Wang et al., 2020). According to a systematic review and meta-analysis on depression, anxiety and stress in China during the COVID-19 pandemic, the prevalence of depression and anxiety was moderately high, and the prevalence of stress was very high (Bareeqa et al., 2020). Some studies have revealed an increase in the loneliness of older adults since the outbreak of COVID-19 (Bao et al., 2021; Dahlberg, 2021). Furthermore, evidence from empirical studies has shown that both loneliness and mental illness are risk factors for cognitive decline (Gulpers et al., 2016; Sussams et al., 2020). This emphasizes that it is necessary to adjust for mental health status when analyzing the association between hearing loss and cognitive impairment, as was done in this longitudinal study.

### Strengths

Non-verbal materials were utilized in this study, both for the general cognitive performance and in specific domains, which may be a better estimation of the association between hearing loss and cognitive impairment than achieved when using verbal materials that are based on verbal materials. It is possible that age-related hearing loss and cognitive impairment share a common etiology, such as vascular disease. Furthermore, this study provides further evidence that the link between hearing loss and cognitive decline may also be mediated by depressive disorders and social isolation. This study attempted to control for these covariates and showed that the associations remained robust.

### Limitations

Two limitations have already been noted above. The short follow-up period may underpower the potential association between audiometric hearing loss and cognitive declines at the scale of general and specific domains. Furthermore, the participants experienced the COVID-19 pandemic between the baseline (2019/2020) and the 12 month follow-up session (2020/2021). At this stage, it is difficult to distinguish to what extent the pandemic impacted the mental health and also cognitive function of these older adults.

Another limitation is that as a volunteer cohort, older adults with high self-awareness of health concerns may have tended to be enrolled; therefore, extrapolating the findings from this current study must be done with caution. More extensive studies with more representative and community-based participants will be required to confirm the results.

Central auditory processing function was not measured in this study. There is evidence that central auditory processing impairment is the primary impairment associated with cognitive impairment and dementia (Sardone et al., 2019). Future studies regarding hearing and cognitive decline in a tonal-language population should also investigate the impact of central auditory processing.

Finally, the recommended cutoff score for the MoCA, 26, was adopted for cognitive impairment in this study, which may result in a higher rate of identification of those at risk of cognitive impairment and dementia, and increase the odds ratio between hearing loss and cognitive impairment (Roalf et al., 2016). Recently, a meta-analysis revealed a cutoff score of 23/30 yielded a better diagnostic accuracy for the identification of a cognitive impairment (Carson et al., 2018).

### Clinical implications

This study contributes to the growing but still limited body of evidence that hearing loss and cognitive impairment, and decline are significantly associated in tonal language-speaking older adults in China. Hearing loss may be a modifiable solution for health problems associated with cognitive impairment. As hearing aids are the primary management option for hearing loss, it is important to conduct a clinical trial to investigate whether hearing aids can delay cognitive decline, especially in a randomized controlled trial (Jayakody et al., 2020).

Given the high occurrence of dementia, actions to reduce these concerns for seniors may have considerable public health implications. Steps should also be taken to incorporate hearing assessment and cognitive screening in clinical protocols for older adults 60 years and above in both hearing and memory clinics. Clinicians should start thinking about incorporating objective electrophysiological measurements as part of the hearing test battery, considering their advantage in evaluating hearing acuity in older adults with cognitive impairment, who are unable to complete behavioral pure tone audiometry (Tarawneh et al., 2022). Furthermore, for hearing-related professionals, little is known about their knowledge, attitude and practice concerning providing care to hearing-impaired individuals with dementia. Studies in this field will be essential to empowering hearing-related professionals to deliver optimal healthcare services to older adults who have both hearing loss and cognitive impairment.

## Conclusion

In summary, this study found that moderate or above hearing loss at baseline was associated with cognitive decline during 12 months of follow-up in a Chinese language-speaking population aged 60 years and above. This finding supplied evidence that hearing loss may be a risk factor for cognitive impairment in older adults. Given that more than 50 per cent of adults older than 60 years in China are affected by hearing impairment, hearing intervention may be a target to prevent or at least delay cognitive decline in older adults.

## Data availability statement

The raw data supporting the conclusions of this article will be made available by the authors, without undue reservation.

## Ethics statement

The studies involving human participants were reviewed and approved by the University of Western Australia-Human Research Ethics Committee (RA/4/20/5538) and Beijing Tongren Hospital, Capital Medical University (TRECKY2019-090). The patients/participants provided their written informed consent to participate in this study.

## Author contributions

DJ and XF designed the experiments. XF carried out the experiments. XF, RE, DJ, SW, and BL analyzed the experimental data. XF wrote the manuscript. RE and DJ revised the manuscript. RE, DJ, SW, and BL reviewed the manuscript. All authors contributed to the article and approved the submitted version.
