# Supplementary material for: The longitudinal relationship between hearing loss and cognitive decline in tonal language-speaking older adults in China
Source: Front Aging Neurosci. 2023 Mar 17;15:1122607. doi: 10.3389/fnagi.2023.1122607 (PMC10063895; doi:10.3389/fnagi.2023.1122607)
Supplement: Supplementary file 1 [file Data_Sheet_1.docx]

# Supplementary materials

The longitudinal relationship between hearing loss and cognitive decline in tonal language-speaking older adults in China

Xinxing Fu, Robert H. Eikelboom, Bo Liu, Shuo Wang, Dona M.P. Jayakody

Figure S-1.

| 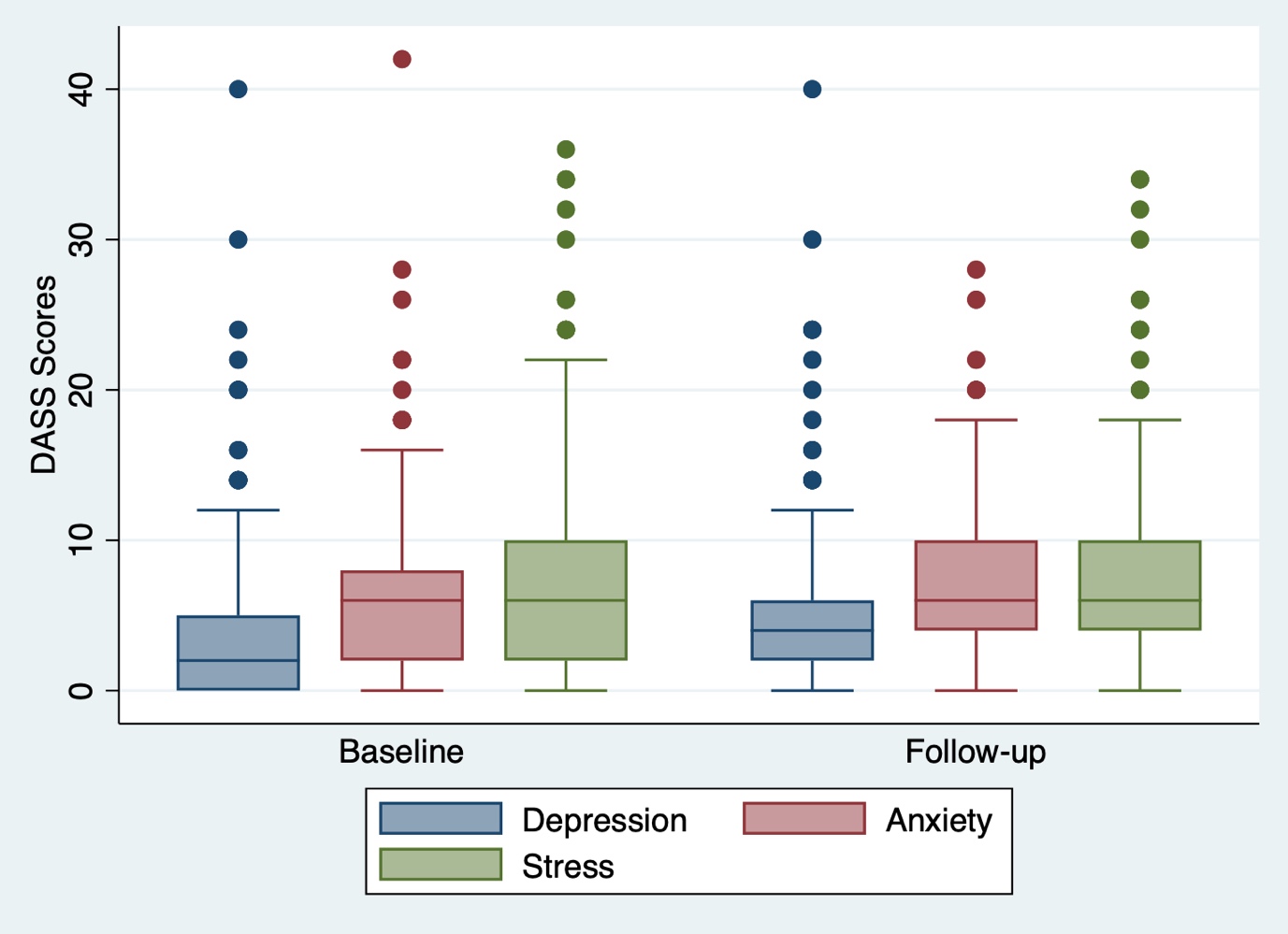 |
| --- |
| Box and whisker plot of DASS depression, anxiety and stress scores at baseline and follow-up. Any outlier at baseline/follow-up is shown by a dot. DASS: Depression Anxiety Stress Scale. |

Figure S-2.

| 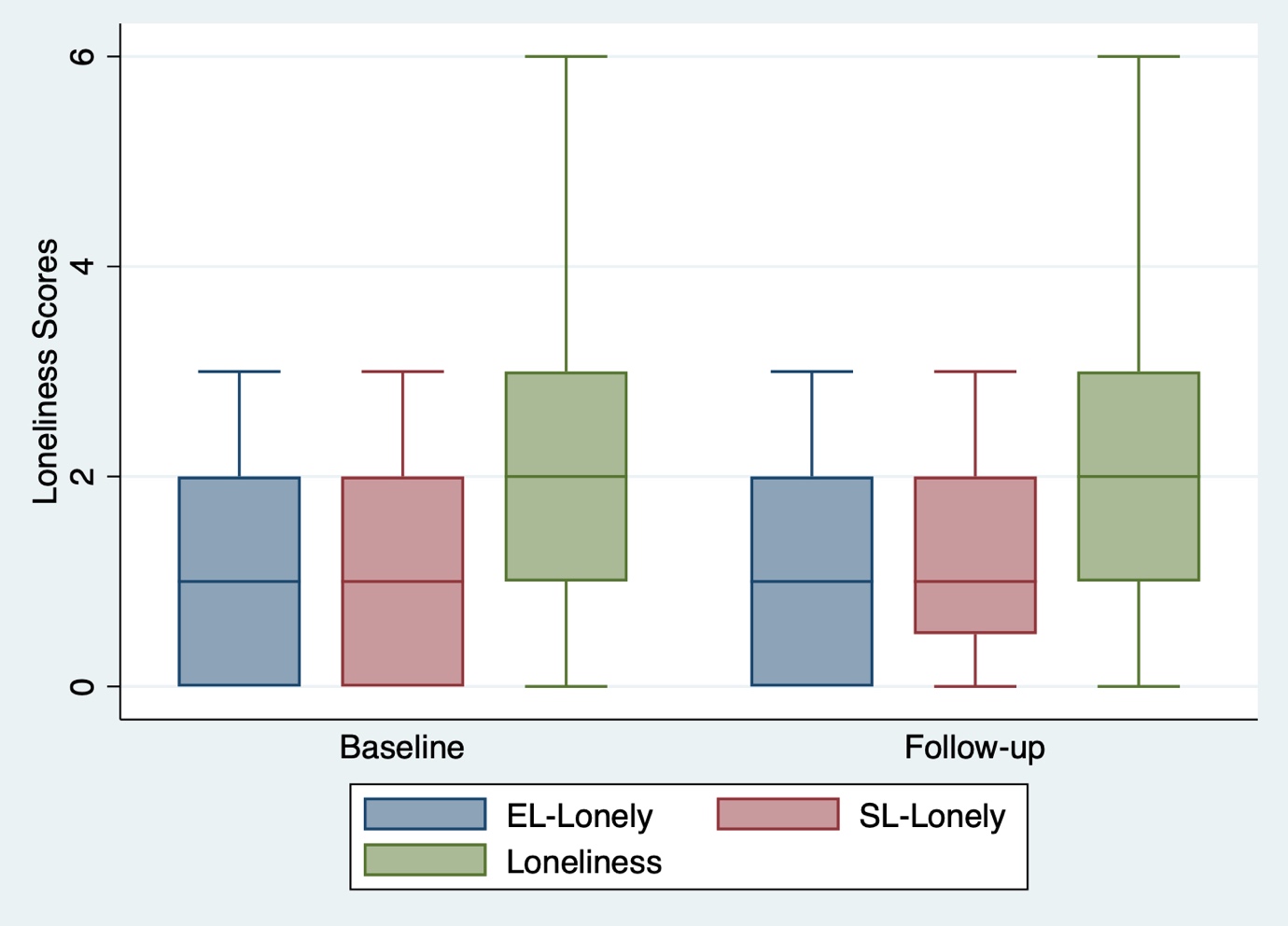 |
| --- |
| Box and whisker plot of loneliness scores at baseline and follow-up. |
